# The impact of age and intensity of treatment on the outcome of traumatic brain injury

**DOI:** 10.3389/fneur.2024.1471209

**Published:** 2024-11-22

**Authors:** Alberto Corriero, Anna Fornaciari, Samuel Terrazzino, Rossella Zangari, Antonio Izzi, Lorenzo Peluso, Marzia Savi, Chiara Faso, Laura Cavallini, Martina Polato, Eva Vitali, Sophie Schuind, Fabio Silvio Taccone, Elisa Gouvêa Bogossian

**Affiliations:** ^1^Department of Interdisciplinary Medicine-Intensive Care Unit Section, University of Bari Aldo Moro, Bari, Italy; ^2^Department of Intensive Care, Hôpital Universitaire de Bruxelles (HUB), Université Libre de Bruxelles (ULB), Brussels, Belgium; ^3^UOC Anesthesia and Intensive Care II, IRCCS Casa Sollievo Della Sofferenza Viale Cappuccini, San Giovanni Rotondo, Italy; ^4^Department of Biomedical Sciences, Humanitas University, Via Rita Levi Montalcini, Milan, Italy; ^5^Department of Anesthesia and Intensive Care, Humanitas Gavazzeni, Bergamo, Italy; ^6^Department of Neurosurgery, Hôpital Universitaire de Bruxelles (HUB), Université Libre de Bruxelles (ULB), Brussels, Belgium

**Keywords:** traumatic brain injury, age, treatment, mortality, neurological outcome, TBI, TIL

## Abstract

**Background:**

Approximately one-third of trauma-related deaths are due to traumatic brain injury (TBI), particularly among young adults and elderly patients. Management strategies may vary across different age groups, potentially influencing short-term neurological outcomes. This study aims to investigate age-related disparities in treatment approaches and 3-month neurological outcomes among TBI patients.

**Methods:**

We conducted a retrospective study on TBI patients requiring Intensive Care Unit (ICU) admission from January 1, 2015, to January 1, 2024, in a tertiary University hospital. Patient demographics, major comorbidities, ICU admission parameters, interventions and ICU complications were collected. An unfavorable neurological outcome at 3 months (UO) was defined as a Glasgow Outcome Scale (GOS) score of 1–3. A high therapy intensity level (TIL) was defined as a TIL basic of 3–4. A multivariable logistic regression model and a Cox proportional Hazard Regression model were used to assess the association of age and TIL with neurological outcome and mortality. A sensitivity analysis on low TIL (0–2) and high TIL subgroups was also conducted.

**Results:**

We enrolled 604 TBI patients, of which 240 (40%) had UO. The highest prevalence of UO was found in patients aged ≥80 years (53/94, 56%), followed by patients aged 50–79 years (104/255, 41%). The age group 35–49 years had the lowest rate of UO (38/127, 30%). Older patients (age ≥ 80 years) received less frequently high TIL than others (*p* = 0.03). In the multivariable analysis, age ≥ 80 years [OR: 3.42 (95% CI 1.72–6.81)] was independently associated with UO, while age ≥ 80 years [HR 5.42 (95% CI 3.00–9.79)] and age 50–79 years [HR 2.03, (95% CI 1.19–3.48)] were independently associated with mortality. Although there was no interaction between age groups and TIL on outcome, an exploratory analysis showed that in the high TIL subgroup of patients, age had no independent impact on the outcome, whereas, in the low TIL group, age ≥ 80 years was independently associated with UO [OR: 3.65 (95% CI: 1.64–8.14)].

**Conclusion:**

Older age, especially in the setting of low intensity treatment, may impact short-term neurological outcome of traumatic brain-injured patients.

## 1 Introduction

Traumatic Brain Injury (TBI) poses a significant health burden worldwide, affecting approximately 70 million individuals annually, regardless of the underlying mechanisms of injury (1). Considering all ages and severities in Europe, the crude incidence of TBI varies from 47.3 per 100,000 to 694 per 100,000 inhabitants in country-level studies (2). TBI exhibits two distinct incidence peaks: in children and young adults (typically aged 18–40 years) and older adults (over 60 years). The first peak primarily results from high-risk behaviors and activities, such as motor vehicle accidents and sports injuries, while the second peak is mainly due to falls, which are more common in the elderly population (3, 4). These age-related patterns underscore the need for targeted prevention strategies to reduce TBI risk in these vulnerable groups (5, 6).

Several studies have revealed that older age is a significant negative prognostic factor after TBI (7–11). These issues justify the need for further research using standardized protocols, comprehensive assessments, longer follow-up periods, and larger sample sizes to understand better how age affects TBI outcomes, ultimately leading to better use of resources and improved patient care. Furthermore, diagnostic testing and treatment interventions may vary among different age groups, and a lower level of management intensity could explain the association between older age and poor outcomes (12). Indeed, elderly TBI patients are less likely to receive the activation of trauma teams and advanced imaging, both crucial for early and accurate diagnosis and intervention (13). This conservative approach extends to critical care procedures, where ventilator support and intracranial pressure (ICP) monitoring decrease significantly with older age (13). Surgical treatments, which can be life-saving, are also less frequently performed in elderly patients. Decompressive craniectomy, in particular, is rarely conducted in individuals over 50 despite similar injury severities observed in younger cohorts (12, 14, 15). This decline in treatment intensity is often driven by assumptions about the frailty and limited recovery potential of older patients, which can lead to poorer outcomes.

However, not all TBI patients can be considered using such assumptions. For instance, if adequate acute and post-acute care is provided, a mild TBI may have a much better outcome than moderate and severe TBI, even when older age is considered (13, 16). In fact, inadequate therapy may lessen the chances of survival and recovery for older TBI patients, blunting the potential recovery of such patients, especially the so-called “younger” elderly (17, 18).

The therapy intensity level (TIL) is a composite score that assesses the graded interventions for treating intracranial pressure (ICP) in TBI, with a higher TIL indicating a higher tier of treatments in managing ICP (19, 20). The TIL evaluation includes different treatments such as sedation, osmotic therapy, hypoventilation, hypothermia, cerebrospinal fluid drainage, and surgery. The rationale for using this score is that interpreting ICP is impossible without understanding the therapy’s efforts (20). While prior studies (7–11) have shown that older age is a significant predictor of poor outcomes in TBI patients, few have examined how varying levels of treatment intensity influence these outcomes, especially in elderly patients. This study addresses this critical gap in the literature by investigating how varying levels of TIL affect neurological outcomes in TBI patients across different age groups.

While it is well established that older age is associated with worse outcomes, our study is one of the first to explore whether high-intensity treatment can mitigate these negative effects, particularly in the elderly. By focusing on the interaction between age and therapy intensity, we offer new insights into how personalized, aggressive treatment can improve outcomes even in patients traditionally considered high-risk due to their age.

## 2 Materials and methods

### 2.1 Study design

A monocentric retrospective cohort study was conducted in the Intensive Care Unit of the Hôpital Universitaire de Bruxelles (HUB), Brussels, Belgium. The local ethics committee approved the study protocol (P2020/252), which waived the need for informed written consent because of its retrospective design and since all interventions were part of the standard patients’ care. The study was performed in accordance with the ethical standards of the Declaration of Helsinki.

### 2.2 Study population

We enrolled all adult (>18 years) patients consecutively admitted to our hospital from January 2015 to December 2023 due to TBI, with an available cerebral CT-scan on admission and who stayed at least 24 h in the ICU. We excluded pregnant women and patients transferred from other hospitals.

### 2.3 Traumatic brain injury management

Our center adheres to the current TBI management guidelines as outlined by the Brain Trauma Foundation (21, 22). The management of elevated ICP in TBI includes general measures, such as elevating the head of the bed, sedation, maintaining normothermia, and fluid resuscitation to ensure adequate cerebral perfusion pressure (CPP). Specific interventions involve hyperosmolar therapy using mannitol or hypertonic saline, cautious hyperventilation for acute ICP crises, cerebrospinal fluid drainage, and barbiturate therapy for refractory cases. Continuous ICP monitoring is essential, with thresholds typically set between 15 and 25 mmHg, and maintaining CPP between 60 and 70 mmHg is crucial. Decompressive craniectomy may be considered for refractory intracranial hypertension, while corticosteroids are generally not recommended due to potential adverse effects.

We utilize a tiered algorithm for treating elevated ICP in severe TBI: tier zero: General clinical management, including basic supportive measures; tier one: sedatives, analgesics, hypertonic solutions, and cerebrospinal fluid (CSF) drainage; tier two: more intensive treatments, including neuromuscular blockade and controlled hyperventilation; tier three: high-dose barbiturate therapy, mild hypothermia, and/or decompressive craniectomy.

This structured approach ensures progressively more aggressive management of ICP, tailored to the patient’s response and the severity of their condition. Although the COVID-19 pandemic led to widespread changes in healthcare delivery across the globe, including reduced in-person consultations and shifts in treatment protocols (23), we found no significant differences in the treatment approaches for TBI patients in our cohort during 2020 compared to previous years. Consequently, data from 2020 were included in the analysis without adjustments for pandemic-related changes.

### 2.4 Data collection and definitions

We collected patients’ comorbidities and demographic data. Patients were divided into four age groups: 18–34 years, 35–49 years, 50–79 years, and ≥ 80 years. Severity scores on admission, including the Sequential Organ Failure Assessment (SOFA) (24), the Glasgow Coma Scale (GCS) (25), and the Marshall score (26), were recorded. Additionally, we collected data on admission glycemia, pre-hospital hypoxemia, hypotension on admission, pupillary light reflex, and the presence of extracranial injuries.

We documented the use of various interventions during the ICU stay, such as mechanical ventilation, osmotic agents, inotropic agents, vasopressors, sedatives, invasive and non-invasive neuromonitoring, and all interventions aimed at controlling intracranial hypertension, including hyperventilation, barbiturates, hypothermia, and decompressive craniectomy. We also recorded medical, neurological, and clinical complications during the ICU stay, such as all forms of shock (e.g., vasopressor therapy for more than 6 consecutive hours and lactate levels >2.0 mmol/L), clinical seizures, and ICP exceeding 20 mmHg for more than 5 min and requiring treatment (e.g., intracranial hypertension).

All interventional variables were used to assess the intensity of ICP treatment to compute the basic TIL; the highest TIL basic for each patient was calculated for any day during the ICU stay. The TIL scale, as defined by Maas et al. (27), in its simplified version, assigns TIL 0 to no specific ICP-directed therapy, TIL 1 to basic ICU care (e.g., sedation, head-up positioning, and normocapnia), TIL 2 to mild therapy (e.g., low-dose osmotic therapy, mild hypocapnia, and CSF drainage), TIL 3 to moderate therapy (e.g., higher dose osmotic therapy, moderate hypocapnia, and higher CSF drainage), and TIL 4 to extreme therapy (e.g., profound hypocapnia, hypothermia, metabolic suppression for ICP control, and surgery for refractory ICP). In our study, a high TIL was defined as TIL 3–4, while a low TIL was defined as TIL 0–2.

TBI severity was categorized using the GCS: mild TBI as a GCS score of 13–15, with loss of consciousness (LOC) up to 30 min and post-traumatic amnesia (PTA) less than 24 h; moderate TBI as a GCS score of 9–12, LOC between 30 min and 24 h, and PTA from 24 h to 7 days; severe TBI as a GCS score of 8 or below, LOC over 24 h, and PTA more than 7 days (28). ICU mortality and hospital mortality were also recorded. Neurological outcome was assessed at 3 months using the Glasgow Outcome Scale (29). An unfavorable neurological outcome was defined as a GOS of 1 to 3 (UO; death, vegetative state, and severe disability, respectively). A favorable neurological outcome was defined as a GOS of 4 or 5 (moderate disability, mild disability or asymptomatic).

### 2.5 Outcome assessment

The study’s primary analysis was to investigate the association between age and UO considering TIL. The secondary analysis of the study was to investigate the association between age and in-hospital mortality, taking into account TIL.

### 2.6 Statistical analysis

We conducted a descriptive statistical analysis using the Medcalc program for Windows v20.217. For continuous variables, data are expressed as mean (±SD) or median and interquartile ranges (IQR), according to the distribution of variables. Categorical variables are described as count and percentage. Categorical variables were compared using the Chi-square or Fisher exact test. Continuous variables were compared using t-Student test or Mann–Whitney test according to their distribution. We performed a univariate and multivariate logistic regression to study the association between the age groups and neurological outcome, adjusted for high intensity of treatment and the following predefined variables which are known factors associated with outcome in the literature: GCS on admission, Marshall CT score on admission, pupillary light reflex on admission, pre hospital/admission hypotension, prehospital/admission hypoxemia, seizures, shock during ICU stay, traumatic subarachnoid hemorrhage. We assessed the interaction between these variables and age groups. We tested all variables for multicollinearity to avoid strong correlations. All variables were tested for multicollinearity before modeling. We also assessed the interaction between age groups and TIL on measured outcomes (*p* value for interaction). We reported odds ratio (OR) and 95% Confidence Intervals (CI) for all variables included in the model. Similarly, we performed a Cox regression analysis to assess the association between age and in-hospital mortality adjusted for high intensity of treatment and for the same predefined variables that have previously been reported in the literature as factors associated with hospital death. We reported hazard ratios (HR) and 95% confidence intervals (CI) for all variables included in the model. As described above, we also performed a exploratory subgroup analysis of patients according to the TIL score.

Furthermore, In the case of our study, logistic regression was selected over Poisson regression due to the binary nature of the primary outcomes (e.g., mortality and neurological outcome). Logistic regression is the standard approach for analyzing binary outcomes and provides easily interpretable odds ratios, which are commonly used in TBI research. While Poisson regression with robust standard errors is appropriate for common outcomes to obtain prevalence ratios, logistic regression remains a well-validated method for binary data and is consistent with the methodology of similar studies in the field, such as in Dhandapani et al. (30). Finally, given the relatively small sample size in certain subgroups (e.g., patients over 80 years old), logistic regression was preferred due to its robustness in handling such data. We acknowledge the potential for odds ratio overestimation, but logistic regression allows for comparability with other TBI studies and remains a valid analytical choice.

## 3 Results

### 3.1 Study population

We identified 623 patients admitted to the ICU due to TBI with an initial cerebral CT-scan showing signs of head trauma, of whom 19 were excluded due to loss of follow-up, for a total of 604 included patients in the final analysis. Among them, 193 (32%) had severe TBI on admission. The mean age of the population was 56 (± 21) years, and the male sex was predominant (65%). Falls (*n* = 427–70%) were the most frequent injury mechanism within all age groups. The median GCS on admission was 14 (6–15). Intracranial hypertension was diagnosed in 191/604 patients (32%) during the ICU stay. The characteristics of the study population are reported in Table 1. A total of 144 (24%) patients died during the hospital stay, and 240 (40%) of patients had unfavorable neurological outcome.

**Table 1:** Characteristics of the study population according to different age groups.

	All patients (*N* = 604)	Age 18–34 N = 129	Age 35–49 *N* = 127	Age 50–79 *N* = 254	Age ≥ 80 *N* = 94	*p*- value
Age (years), mean(SD)	55 (21)	27 (5)	42 (5)	66 (9)	86 (4)	<0.001
Males, n (%)	397 (66)	96 (74)	102 (80)	158 (62)	41 (44)	<0.001
High TIL, n (%)	144 (24)	34 (26)	37 (29)	60 (24)	13 (14)	0.03
TIL BASIC, median (IQR)	1 (0–2)	1 (0–3)	1 (0–3)	1 (0–2)	1 (0–2)	0.23
Mechanisms						
Fall, *n* (%)	427 (70)	56 (43)	77 (61)	205 (81)	89 (95)	<0.001
Motor vehicle accident, *n* (%)	12 (2)	5 (4)	3 (2)	4 (2)	0	0.21
Violence, *n* (%)	38 (6)	14 (11)	16 (13)	7 (3)	1 (1)	<0.001
Unclear mechanism	127 (21)	54 (42)	31 (24)	38 (15)	4 (4)	<0.001
Comorbidities						
Hypertension, *n* (%)	194 (32)	5 (4)	9 (7)	123 (48)	57 (61)	<0.001
Diabetes, *n* (%)	70 (12)	0 (0)	6 (5)	47 (18)	17 (18)	<0.001
Previous heart disease, *n* (%)	132 (22)	1 (1)	6 (5)	79 (31)	46 (49)	<0.001
Previous neurologic disease, *n* (%)	119 (20)	3 (2)	4(3)	72 (28)	40 (43)	<0.001
Chronic kidney disease, *n* (%)	41 (7)	0 (0)	1 (1)	26 (10)	14 (15)	<0.001
COPD, *n* (%)	39 (6)	2 (2)	3(2)	24 (9)	10 (11)	0.002
Immunosuppression, *n* (%)	16 (3)	1 (1)	2 (2)	12 (5)	1 (1)	0.06
Cancer, *n* (%)	49 (8)	0 (0)	3 (2)	39 (15)	7 (7)	<0.001
Liver cirrhosis, *n* (%)	15 (2)	0 (0)	0 (0)	13 (5)	2 (2)	0.003
Alcohol, *n* (%)	172 (28)	37 (29)	52 (41)	78 (31)	5 (5)	<0.001
Smoking, *n* (%)	76 (13)	26 (20)	23 (18)	25 (10)	2 (2)	0.001
On admission						
SOFA score, median (IQR)	3 (0–6)	2 (0–6)	2 (0–7)	3 (1–7)	3 (1–6)	0.34
Polytrauma, *n* (%)	214 (35)	64 (50)	56 (44)	72 (28)	22 (23)	<0.001
Both pupils reacting, *n* (%)	506 (84)	107(83)	107 (84)	219 (86)	73 (78)	0.29
GCS, median (IQR)	14 (6–15)	13 (5–15)	13 (4–15)	14 (8–15)	13 (6–15)	0.48
Severe TBI, *n* (%)	193 (32)	47 (36)	49 (39)	66 (26)	31 (33)	0.05
Marshall score, median (IQR)	2 (2–5)	2 (2–5)	2 (2–5)	2 (2–5)	2 (2–3)	0.48
Traumatic SAH on CT-scan, *n* (%)	319 (53)	62 (48)	70 (55)	138 (54)	49 (53)	0.64
Epidural hematoma on CT-scan, *n* (%)	341 (56)	63 (49)	67 (53)	153 (60)	58 (62)	0.10
Sodium (mmol/l),median (IQR)	139 (108–157)	140 (2–4)	139 (137–142)	138 (135–141)	138 (135–141)	0.004
Glucose (mg/dl), median (IQR)	128 (108–157)	120 (106–139)	123 (104–147)	130 (108–162)	142 (118–169)	0.001
Hemoglobin (g/dl), median (IQR)	13 (12–14)	14 (12–15)	14 (13–15)	13 (11–14)	12 (12–14)	0.001
Pre hospital/admission hypotension, *n* (%)	90 (15)	18 (14)	19 (15)	46 (18)	7 (7)	0.10
Pre- hospital hypoxemia, *n* (%)	170 (28)	30 (23)	40 (32)	72 (28)	28 (30)	0.50
During ICU stay						
Vasopressors, *n* (%)	202 (33)	46 (36)	43 (34)	91 (36)	22 (23)	0.16
Inotropic agents, *n* (%)	12 (20)	2 (2)	3 (2)	5 (2)	2 (2)	0.97
Mechanical ventilation, *n* (%)	303 (50)	62 (48)	69 (54)	129 (51)	43 (46)	0.60
RRT, *n* (%)	8 (1)	0 (0)	1 (1)	6 (2)	1 (1)	0.25
ICP monitoring, *n* (%)	156 (26)	42 (24)	40 (32)	65(26)	9 (10)	0.001
PbtO_2_ monitoring, *n* (%)	56 (9)	16 (12)	17 (13)	20 (8)	3 (3)	0.03
Sedatives, *n* (%)	268 (44)	61 (47)	59 (56)	112 (44)	36 (38)	0.56
Opioids, *n* (%)	357 (59)	79 (61)	82 (65)	143 (56)	53 (56)	0.40
Osmotic therapy, *n* (%)	178 (30)	46 (36)	44 (36)	69 (27)	19 (20)	0.04
Decompressive craniectomy, *n* (%)	71 (12)	17 (13)	22 (17)	29 (11)	3 (3)	0.01
Barbiturates, *n* (%)	39 (6)	15 (12)	15 (12)	9 (4)	0 (0)	0.001
Hypothermia (<35°C), *n* (%)	18 (3)	7 (5)	7 (6)	4(2)	0 (0)	0.02
Intracranial hypertension, *n* (%)	191 (32)	46 (36)	46 (36)	79 (31)	20 (21)	0.08
Seizures, *n* (%)	71 (12)	11 (8)	10 (8)	36 (14)	14 (15)	0.14
Shock, *n* (%)	37 (6)	6 (5)	10 (8)	17 (7)	4 (4)	0.60
Red blood cells transfusions, *n* (%)	106 (18)	21 (16)	21 (16)	50 (20)	14 (15)	0.67
Outcomes						
ICU stay, days - median (IQR)	3 (2–7)	3 (2–8)	3 (2–8)	3 (2–7)	3 (2–3)	0.02
Hospital stay, days - median (IQR)	12 (5–28)	8 (4–28)	12 (5–30)	13 (5–30)	10 (3–23)	0.11
GOS at 3 months, (points), median (IQR)	4 (2–5)	4 (3–5)	4 (3–5)	4 (1–5)	3 (1–4)	<0.001
Unfavorable outcome, *n* (%)	240 (40)	45 (35)	38 (30)	140 (41)	53 (56)	0.001
In-hospital death, *n* (%)	144 (24)	18 (14)	20 (16)	64 (25)	42 (45)	<0.001

Data are presented as mean (±SD), count (%) or median (25th–75th percentiles).

### 3.2 Characteristics of the population according to different age groups

The characteristics of the study population according to different age groups are described in Table 1. The majority of patients (254, 42%) were aged between 50 and 79 years. The median GCS score on admission, Marshall score, and the percentage of severe TBI were not significantly different between the age groups. The mean TIL basic was also not different between groups; however, patients aged ≥80 years had a lower prevalence of high TIL compared to other groups. Additionally, patients aged ≥80 years, as well as those aged 50–79 years, had higher mortality rates than younger patients. Furthermore, patients aged ≥80 years had a higher rate of UO compared to the other age groups.

### 3.3 Age groups and neurological outcome

Patients with UO were older, had lower GCS scores on admission, a higher incidence of organ dysfunction, and received a higher TIL compared to those with favorable outcomes (Supplementary Table S1). Furthermore, epidural hematoma and traumatic subarachnoid hemorrhage were more frequently present in the UO group. In the multivariate logistic regression model, age ≥ 80 years [OR 3.42 (95% CI 1.72–6.81)] was independently associated with UO (Table 2). There was no interaction between age and TIL groups on UO (High TIL* age 35–49 years, *p* = 0.47; High*TIL age 50–79 years, *p* = 0.98; High TIL* age ≥ 80 years, *p* = 0.87).

**Table 2:** Univariate and multivariate logistic regression of factors associated with unfavorable neurological outcome at 3 months.

	Univariate analysis OR (95% CI)	Multivariate analysis OR (95% CI)
Age 35–49 years	0.56 (0.35–0.86)	0.59 (0.29–1.19)
Age 50–79 years	1.09 (0.78–1.52)	1.66 (0.93–2.96)
Age ≥ 80 years	2.23 (1.43–3.49)	3.42 (1.72–6.81)
Both pupils reacting	0.05 (0.03–0.10)	0.22 (0.10–0.49)
GCS on admission	0.79 (0.76–0.82)	0.88 (0.83–0.93)
High TIL score	6.48 (4.26–9.85)	1.73 (0.95–3.13)
Marshall CT-scan score	1.77 (1.57–1.99)	1.22 (1.04–1.43)
Prehospital/admission hypotension	6.15 (3.67–10.28)	1.48 (0.72–3.04)
Prehospital/admission Hypoxemia	4.98 (3.4–7.28)	1.31 (0.75–2.27)
Seizures	1.77 (1.08–2.91)	1.55 (0.84–2.86)
Shock	11.05 (4.24–28.79)	3.84 (1.24–11.90)
Traumatic SAH on CT-scan	1.98 (1.42–2.76)	1.31 (0.86–2.01)

TIL, treatment intensity level; GCS, Glasgow coma scale; SAH, subarachnoid hemorrhage. Goodness of fit: *p* = 0.995. Values are presented as odds ratio (OR) and 95% confidence interval (CI). Age 18–34 years was used as reference. The interaction between High TIL and age groups was checked and was not statistically significant; therefore, TIL was not included in the final model.

### 3.4 Age groups and in-hospital mortality

**Figure 1:**
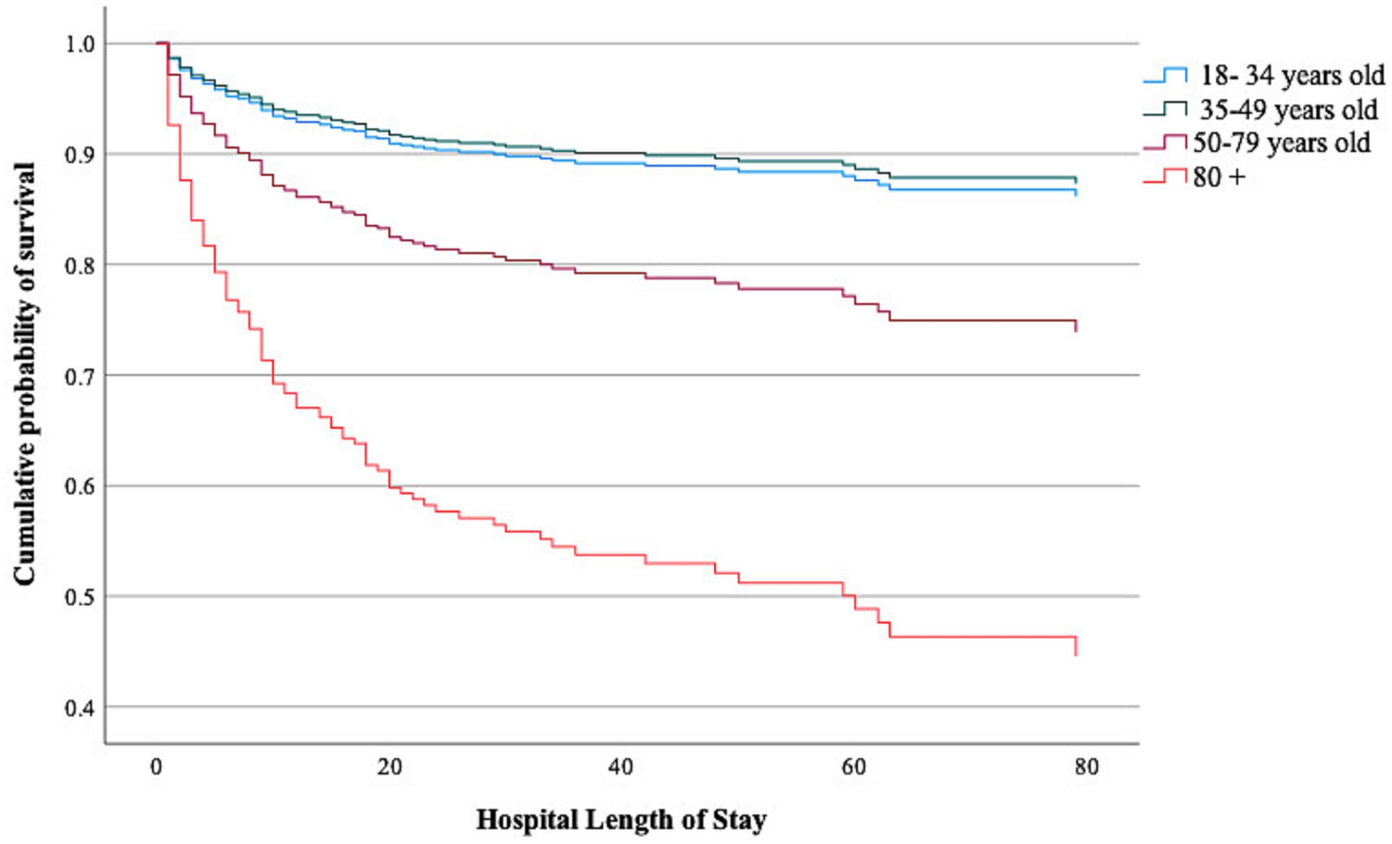
Cumulative incidence of survival over time according to age groups. *p*-value was calculated using a Cox proportion hazard regression model adjusted for High TIL, GCS on admission, pupil reactivity on admission, Marshall CT score on admission, Prehospital/admission hypotension, Prehospital/admission hypoxemia, traumatic SAH.

Non-survivors were older, had lower GCS scores, worse Marshall scores, a higher frequency of hypoxemia and hypotension on admission, and received a higher TIL compared to survivors (Supplementary Tables S2, S3). In an adjusted Cox proportional hazard regression analysis, we found that age ≥ 80 years [HR 5.42 (95% CI 3.00–9.79)] and the age group 50–79 years [HR 2.03 (95% CI 1.19–3.48)] were both associated with a higher risk of in-hospital death (Table 3 and Figure 1). There was no interaction between age and TIL groups on in-hospital mortality (High TIL* age 35–49 years, *p* = 0.47; High*TIL age 50–79 years, *p* = 0.08; High TIL* age ≥ 80 years, *p* = 0.08).

**Table 3:** Multivariate cox proportional hazard regression analysis of factors associated with in hospital death.

	Multivariate analysis HR (95% CI)
High TIL score	1.32 (0.67–1.60)
Age 35–49 years	0.91 (0.48–1.73)
Age 50–79 years	2.03 (1.19–3.48)
Age ≥ 80 years	5.42 (3.00–9.79)
Both pupils reacting	0.33 (0.21–0.5)
GCS on admission	0.88 (0.84–0.93)
Marshall score	1.28 (1.12–1.46)
Prehospital/admission hypotension	0.88 (0.56–1.39)
Prehospital/admission Hypoxemia	0.98 (0.65–1.49)
Seizures	1.12 (0.69–1.81)
Shock during ICU stay	2.02 (1.26–3.24)
Traumatic SAH on CT-scan	0.91 (0.63–1.29)

TIL, treatment intensity level; GCS, Glasgow coma scale; SAH, subarachnoid hemorrhage. HR, Hazard Ratio; CI, confidence interval.

### 3.5 Exploratory subgroup analysis

Patients with high TIL (*n* = 144, 24%) were younger, had more organ dysfunction, higher Marshall CT scores, lower GCS on admission, and worse neurological outcomes compared to others (Supplementary Table S4). In the high TIL subgroup, multivariate logistic regression analysis found no association between age and UO (Supplementary Table S5). In the low TIL group, age ≥ 80 years [OR 3.65 (95% CI 1.64–8.14)] was independently associated with UO (Supplementary Table S6). Similarly, a multivariate Cox proportional hazard regression analysis found that in the high TIL subgroup, age was not associated with in-hospital mortality (Supplementary Table S7), while age 50–79 years [HR 6.31 (95% CI 1.83–21.75)] and age ≥ 80 years [HR 21.25 (95% CI 5.88–76.74)] were independently associated with in-hospital mortality in the low TIL group (Supplementary Table S8).

## 4 Discussion

In this study, we found that age ≥ 80 years was independently associated with worse neurological outcomes and that age ≥ 50 years was associated with increased in-hospital mortality. However, in a exploratory hypothesis generating subgroup analysis considering patients who received high-intensity treatment, age did not increase the likelihood of poor outcomes at 3 months or in-hospital mortality. In contrast, among patients who received lower-intensity therapy, age ≥ 80 years was independently associated with poor outcomes and age ≥ 50 years was independently associated with in-hospital mortality.

Currently, the rate of hospital admissions due to TBI is higher in the elderly population (≥ 65 years old) than in other age groups (31). This patient population is more susceptible to falls, which usually cause mass effect injuries, and they have more comorbidities and severe baseline cognitive or functional deficits (32), increasing the risk of post-recovery functional decline compared to younger patients (33). Indeed, older age has consistently been reported as a factor associated with unfavorable outcomes (34–37) and mortality (33, 38–40) in this setting. However, this association may be impacted by frailty, which represents the cumulative decline in physiological systems over a lifetime (13), making patients more vulnerable to further insults such as TBI and susceptible to poorer outcomes. A recent study has shown that older patients have higher frailty scores, and frailty was an independent determinant of poor outcomes (41). Previous studies have also demonstrated that older TBI patients, especially those without significant pre-existing comorbid conditions (42–44), can survive and recover, reinforcing that factors other than age are important when prognosticating these patients (14, 18, 45). Moreover, a study from the CENTER-TBI database recently highlighted that patients receiving high-intensity treatments may have better outcomes despite the initial severity of the illness (46). This finding has also been confirmed in the SYNAPSE-ICU study, which showed that ICP monitoring and higher TIL were associated with lower mortality, despite older patients not being specifically analyzed in detail for outcomes, based on age groups (47). Similarly, Skaansar et al. demonstrated that a low management intensity strategy correlated with higher 30-day mortality in elderly patients (12).

Despite these findings, older TBI patients are less frequently admitted to the ICU, receive less intensive monitoring (such as invasive ICP), and undergo fewer life-saving interventions like mechanical ventilation or surgery, as highlighted in previous studies (11, 48–51). For instance, randomized clinical trials of surgical interventions in TBI, such as decompressive craniectomy, often exclude patients over the age of 60–65 years (52, 53). The clinical implications of this study, particularly for elderly patients, deserve close attention. Aggressive interventions such as decompressive craniectomy in this population carry inherent risks due to frailty, pre-existing comorbidities, and polypharmacy. These factors likely contribute to the exclusion of older patients from clinical trials, as studies have demonstrated higher mortality and complication rates in this age group (52, 53). Frailty and functional status play a pivotal role in the recovery potential of elderly patients and must be carefully balanced against the potential benefits of invasive procedures (54–56). Geriatric trauma outcome scores (GTOS) and tools like the GERtality Score can help quantify these risks (54–56). These scoring systems, validated in predicting mortality and poor outcomes in elderly trauma patients, aid clinicians in identifying which patients might benefit from high-intensity treatments (57). Additionally, socioeconomic status (SES) has been shown to significantly influence healthcare access, with lower SES often correlating with poorer outcomes due to limited access to post-acute care and rehabilitation services (58).

Additionally, patients included in the BEST-TRIP randomized clinical trial that assessed the impact of ICP monitoring on the outcome of TBI patients were young, with a median age of 29 years, despite not having an upper age limit as an exclusion criterion, further demonstrating that elderly patients are often not offered specific modalities of monitoring and treatment (59). Nevertheless, successful rehabilitation and community reintegration are possible for older TBI patients when adequately treated (49, 60–62).

When assessing the prognosis of brain-injured patients, a so-called “self-fulfilling prophecy” occurs, e.g., when a comatose patient is given a poor prognosis, leading to the withholding of life-sustaining care based on that prediction, which directly results in the patient’s poor outcome (e.g., death) (63). This phenomenon is common in the elderly population but can also be observed in younger patients. A recent study found that among 1,400 patients with severe TBI, some who were taken off life support may have survived and regained some level of independence a few months later, as predicted by a complex mathematical model (64). Neurological recovery and improvement of disorders of consciousness require time and intensive rehabilitation (65) in both elderly and young patients (66). This situation points to a cyclical, self-fulfilling prophecy where clinicians predict poor outcomes based on previously published data in the context of limited therapy, including ICU admission for cases deemed unsalvageable. This assumption leads to withdrawing life support, which increases the likelihood of poor outcomes and further decisions to remove life support (64). In our exploratory analysis, the differences between high and low TIL subgroups may suggest a benefit in treating implementing high intensity treatment to an elderly population. However, this was an exploratory analysis of a retrospective registry and we are unable to ascertain what were the criteria used to decide the intensity of treatment applied to some elderly patients but not others, which may have introduced bias. Further studies are therefore required to investigate this hypothesis. In fact, whether a more proactive therapeutic approach could potentially decrease the high mortality rate and improve neurological outcomes among older TBI patients remains to be validated.

One might speculate that high-tier treatments for TBI patients are costly and not generally worth it compared to comfort care. However, this hypothesis has been contradicted by a study in which researchers developed a decision-analytical model to compare aggressive care, routine care, and comfort care. The study showed that aggressive care may be significantly less expensive up to the age of 80, after which it becomes more expensive than routine care (67). Nonetheless, even for 80-year-old patients, aggressive care may be reasonable depending on the patient’s baseline functional status. Although the *p*-value for interaction was not significantly different when analyzing the impact of age and TIL on measured outcomes, subgroup analyses were performed, as suggested in previous publications (68, 69). These analyses suggested the importance of age on outcomes in relation to the intensity of therapies used to lower ICP.

The advantage of our research lies in its detailed examination of how TIL interacts with age to influence outcomes in TBI patients. While many studies have considered age an independent predictor of worse outcomes, few have explored the mitigating effects of aggressive treatment, especially in older populations. Our findings indicate that high-intensity treatment can improve neurological outcomes and survival even in elderly patients traditionally considered high-risk due to their age. These results challenge conservative treatment practices and suggest that age should not be the sole factor in determining treatment intensity. This study’s focus on the interaction between age and TIL provides new insights into optimizing care for elderly TBI patients and supports a more personalized, aggressive treatment approach.

Our study has several limitations. The retrospective design may introduce biases and limit the ability to establish causal relationships between the identified predictors and clinical outcomes. Additionally, it restricted our analysis to short-term outcomes, which are not ideal for the neurocritical population. Despite our large sample size, the results may lack generalizability as it is a single-center study. Furthermore, the assessment of TIL may be subject to variability in measurement techniques and lacks standardized cut-offs for defining high and low TIL subgroups. The TIL score refers primarily to the intensity of treatment focused on intracranial hypertension, which, while an essential cause of mortality and unfavorable outcomes in TBI patients, does not account for other complications, such as hospital-acquired infections that could have impacted our results. Importantly, we did not evaluate the economic burden associated with patient care across different age groups. Such an analysis requires specialized expertise and falls beyond the scope of our study. Finally, we were unable to assess mortality due to withdrawal of care, which may have influenced our findings, and we did not retrieve the SES of patients. Additionally, we did not evaluate patients’ functional status before admission or their frailty score. Our study also had a limited number of patients aged 80 years or more, which may have reduced the power of the study to detect significant outcomes in this age group and adequately evaluate the interaction between age categories and TIL.

## 5 Conclusion

Our findings suggested that while advanced age is independently associated with worse outcomes in TBI patients, treatment intensity can modify this relationship. High-intensity therapy could lead to better outcomes, even in patients aged 80 years or older, suggesting that age alone should not be a limiting factor in treatment decisions. A personalized, potentially aggressive approach to improve survival and neurological outcomes across age groups, particularly for elderly needs to be further investigated.

## Data Availability

The raw data supporting the conclusions of this article will be made available by the authors, without undue reservation.
